# Dissociated deficits of anticipated and experienced regret in at-risk suicidal individuals

**DOI:** 10.3389/fpsyt.2023.1121194

**Published:** 2023-03-09

**Authors:** Hui Ai, Lian Duan, Lin Huang, Yuejia Luo, André Aleman, Pengfei Xu

**Affiliations:** ^1^Institute of Applied Psychology, Tianjin University, Tianjin, China; ^2^Academy of Medical Engineering and Translational Medicine, Tianjin University, Tianjin, China; ^3^Shenzhen Key Laboratory of Affective and Social Neuroscience, Center for Brain Disorders and Cognitive Sciences, Shenzhen University, Shenzhen, China; ^4^Beijing Key Laboratory of Applied Experimental Psychology, National Demonstration Center for Experimental Psychology Education (BNU), Faculty of Psychology, Beijing Normal University, Beijing, China; ^5^Center for Emotion and Brain, Shenzhen Institute of Neuroscience, Shenzhen, China; ^6^Section Cognitive Neuroscience, Department of Biomedical Sciences of Cells and Systems, University Medical Center Groningen, University of Groningen, Groningen, Netherlands

**Keywords:** suicide, regret, counterfactual thinking, computational modeling, at-risk youths

## Abstract

**Backgrounds:**

Decision-making deficits have been reported as trans-diagnostic characteristics of vulnerability to suicidal behaviors, independent of co-existing psychiatric disorders. Individuals with suicidal behaviors often regret their decision to attempt suicide and may have impairments in future-oriented processing. However, it is not clear how people with suicidal dispositions use future-oriented cognition and past experience of regret to guide decision-making. Here, we examined the processes of regret anticipation and experience in subclinical youth with and without suicidal ideation during value-based decision-making.

**Methods:**

In total, 80 young adults with suicidal ideation and 79 healthy controls completed a computational counterfactual thinking task and self-reported measures of suicidal behaviors, depression, anxiety, impulsivity, rumination, hopelessness, and childhood maltreatment.

**Results:**

Individuals with suicidal ideation showed a reduced ability to anticipate regret compared to healthy controls. Specifically, suicidal ideators’ experience of regret/relief was significantly different from that of healthy controls upon obtained outcomes, while their disappointment/pleasure experience was not significantly different from healthy controls.

**Conclusion:**

These findings suggest that young adults with suicidal ideation have difficulty predicting the consequences or the future value of their behavior. Individuals with suicidal ideation showed impairments in value comparison and flat affect to retrospective rewards, whereas individuals with high suicidality showed blunted affect to immediate rewards. Identifying the counterfactual decision-making characteristics of at-risk suicidal individuals may help to elucidate measurable markers of suicidal vulnerability and identify future intervention targets.

## Introduction

Suicide is the second leading cause of death among adolescents (1), and nearly one-third of suicides occur among young people (2). Heterogeneous risk factors including early-life adversity, psychopathology, and stressful life events can increase suicide risk (3). Given the multifactorial nature of its etiology, which research has yet to fully elucidate, it is difficult to predict suicidal behavior. For example, although major depressive disorder and substance use have been reported as important risk factors for suicide (4), many patients with these conditions do not exhibit suicidal behavior. The assessment of suicide risk is largely dependent on individuals’ self-perception and willingness to report suicidal behaviors (5). Furthermore, adequate intervention for these psychopathologies may not prevent suicide *per se*. It has been proposed that suicidal behavior is an endophenotype that should be studied and treated independently of specific psychiatric disorders (6, 7). Identifying specific risk factors for suicidal behaviors is the first step toward early detection and prevention in at-risk individuals (8).

Decision-making alterations have been found not only in patients with suicidal behaviors related to mood disorders (7, 9–14), but also in suicidal patients with PTSD (15) and schizophrenia [reviewed by (16)]. Moreover, psychiatric patients with suicidal behaviors have shown distinctive impairments during decision-making compared to patients without suicidal behaviors (7, 17). These findings suggest that altered decision-making may be a potential trans-diagnostic marker of suicide, independent of co-existing psychopathologies (8, 18). Although studies on decision-making in suicide have mostly focused on past experiences, assessing reactions to future events may be important for the early detection of suicide (19). Previous studies have shown that suicide attempters differ from non-attempters in future-oriented cognition, characterized by overestimating negative future events and forecasting less happiness for positive future events (19, 20). However, it is unclear how this future-oriented cognition affects present decision-making in suicidal individuals, or whether they are able to use prospective outcomes to guide action selection. Identifying the future-oriented decision-making in suicidal individuals without a diagnosis of psychiatric disorder will help to uncover the cognitive mechanism of suicide independent of diagnosis.

Suicidal individuals often regret their decision to engage in suicidal behavior (21), which involves counterfactual decision-making as well as a regret response. Counterfactual thinking refers to thoughts that compare possible outcomes of alternative choices in the past with the current situation, which often occurs in goal-directed decision-making and coexists with the experience of regret (22). While people generally avoid regret by choosing the option with the least expected regret (23), it has been proposed that suicidal individuals are overly sensitive to self-blamed regret about past events, which may expose them to intense internal conflict and trigger suicidal behavior (24). However, it is unclear whether suicidal individuals have deficits in anticipating or experiencing regret for future events.

Elucidating the future-oriented decision-making and affective processing mechanisms in suicidal at-risk individuals is important for understanding the progression of suicidal behaviors and is the first step toward early detection. Therefore, the aim of our study was to investigate the regret processing in suicide by using a counterfactual thinking paradigm in suicidal at-risk youth without psychiatric disorders. This paradigm has been well-designed to observe value-based predictive behavior as well as emotional responses to counterfactual outcomes (25, 26). Given that suicidal behavior has been reported to be associated with impairments in emotion processing (27), and that healthy individuals tend to be regret-avoidant (28), we predict that individuals with suicidal ideation will show altered emotional responses to outcomes compared to non-suicidal controls. We also predict that suicidal individuals would show impaired performance during counterfactual decision-making since the expected reward value has been found to be disrupted in previous studies of suicidal behavior (10, 29).

## Methods and materials

### Participants

Participants completed the Scale for Suicidal Ideation [SSI-19; (30)]. The SSI-19 is a 19-item scale designed to measure suicidal ideation or intent. Current suicidal ideation in the last 2 weeks and suicidal ideation at the worst point in life were assessed. For those with suicidal ideation, an explicit question on suicide attempts was asked to assess whether they had ever attempted suicide. Because of the high comorbidity between suicidality and psychiatric conditions such as depression and anxiety, participants were instructed to complete the Beck Depression Inventory-II [BDI-II; (31)] and Spielberger’s State–Trait Anxiety Inventory (32). Participants with depressive states (BDI scores above 14) were excluded. The suicidal ideation and control groups were matched for level of state anxiety. Moreover, to control for the possible confounding effects of childhood maltreatment, rumination, hopelessness, and impulsivity (5), participants completed the Childhood Trauma Questionnaire (33), the Rumination Reconsidered scales (34), the Beck Hopelessness Scale (35), and the Barratt Impulsiveness Scale (36). Participants with a diagnosis or family history of mental disorders were excluded from our study. They were also screened with the exclusion criteria of alcohol or substance use and any history of neurological illness.

### Task paradigm

**Figure 1:**
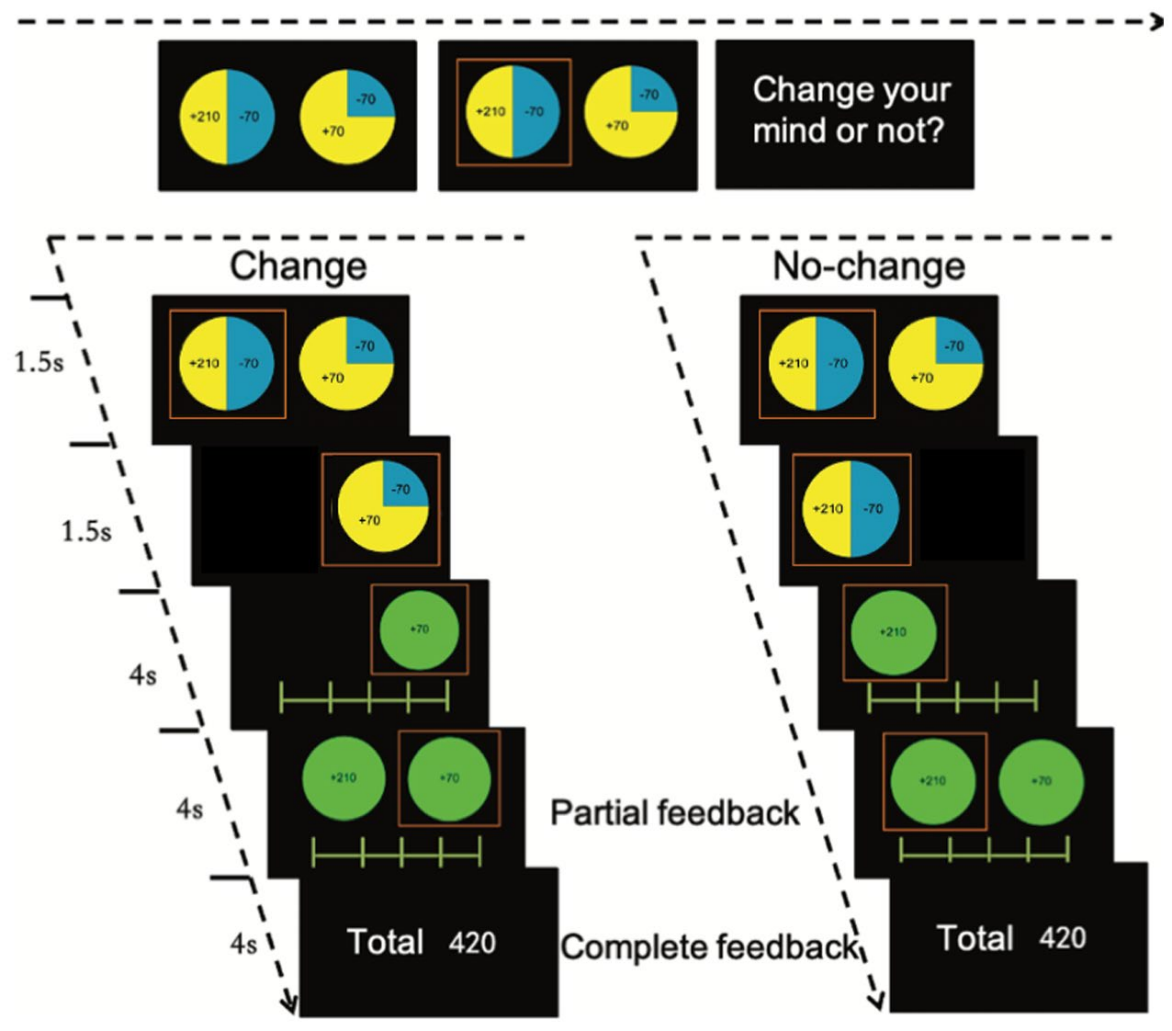
Task procedure. The proportion of two colors in one panel represents the probability of two outcomes. Participants were asked to make a choice and have a 50% opportunity to change their mind. Then, they were asked to rate how they feel after they make the choice (first rating) and after the presentation of the outcomes of the other panel (second rating).

The current counterfactual-thinking task was adapted from those of Baskin-Sommers et al. (37), Gillan et al. (38), and Camille et al. (26). Prior to the experiment, participants were instructed to maximize their score in order to receive more rewards. On each trial, participants were asked to choose one out of two wheels (Figure 1). The proportions of different colors (0.25, 0.5, or 0.75) represented the probability of getting the particular points. There were 16 possible outcomes for each option: −210, 210; −210, 70; −210, −70; −210, −210; −70, 210; −70, 70; −70, −70; −70, −210; 70, 210; 70, 70; 70, −210; 210, 210; 210, 70; 210, −70; 210, −210. To control for between-subject differences in the presentation of trials, each participant received the same order of trials as the others and the probability of outcomes was not randomized.

To exacerbate the regret effect (26), participants had the opportunity to change their mind in 50% of the trials. Once the participant had chosen one of the two wheels, the unchosen wheel was darkened and the chosen wheel was highlighted. After the outcome was presented, a 9-point rating scale appeared on the screen asking participants to rate how they felt about the outcome of the chosen option. The aim of this rating was to assess the emotional experience of counterfactual thinking in relation to achieving another outcome within the same wheel. Following this partial feedback, the outcome of the unchosen option was presented and participants were asked to rate their feelings on a second 9-point rating scale as complete feedback. This rating was designed to measure the emotion resulting from counterfactual thinking on what would have happened if the other option wheel had been chosen. After completing all 80 trials, the participant’s final score was presented on the screen. We used Psychotoolbox-3^1^ to present the stimuli and record the behavioral responses.

### Data analysis

#### Emotion rating scores

For the first rating on partial feedback, we calculated the obtained outcome and the difference between the obtained and unobtained outcomes in the same wheel ({obtained outcome > unobtained outcome of the same wheel} was operationalized as chance counterfactual, indicating the differences between the obtained value and what the participant could have obtained within the chosen wheel). For the second rating on complete feedback, we calculated the obtained outcome and the difference between the obtained and unobtained outcomes in the other wheel ({obtained > unobtained outcome in the other option} was operationalized as agent counterfactual, indicating the differences between the obtained value and what the participant could have obtained if chosen the other wheel). After this, we built linear mixed effect models with lme4 package in R (version 3.6.2) for two rating outcomes with the groups (suicide, control), outcomes of each trail, and chance counterfactuals as fixed-effect predictors, group × chance counterfactual and group × obtained outcome as interaction terms, and participants as a random factor in rating model 1; with group, outcome of each trial, agent counterfactuals as fixed-effect predictors, participants as a random factor, and group × obtained outcome, group × agent counterfactuals as interaction terms in rating model 2.

#### Option-selection modeling

We modeled the counterfactual behaviors by estimating the following three factors guiding decision-making: expected value (EV), expected disappointment (ED), and expectation of regret/relief (regret/relief, R). Here, x_1_ and y_1_ represent two possible outcomes of option 1 and x_1_ > y_1_; x_2_ and y_2_ represent two possible outcomes of option 2, and x_2_ > y_2_; p and 1-p represent the possibilities of obtaining x_1_ and y_1_; q and 1-q represent the probability to get x_2_ and y_2_ in option 2.

With these parameters, we first calculated the maximal expected value with Eq. (1), where EV > 0 indicates a higher EV in option 1 than in option 2.

(1) $$\begin{array}{l} \mathrm{EV}={\mathrm{EV}}_{o1}-{\mathrm{EV}}_{o2}=\left[p*x_{1}+\left(1-p\right)*y_{1}\right]\\ -\left[q*x_{2}+\left(1-q\right)*y_{2}\right] \end{array}$$

We then calculated the expected disappointment (ED) of each trial with Eq. (2), where ED_o1_ and ED_o2_ represent the estimate of expected disappointment for option 1 and option 2, and EDo_2_ > EDo_1_ indicates the participant should choose option 1 when trying to avoid future disappointment.

(2) $$\mathrm{ED}={\mathrm{ED}}_{o2}-{\mathrm{ED}}_{o1}=\left(x_{2}-y_{2}\right)\left(1-q\right)-\left(x_{1}-y_{1}\right)\left(1-p\right)$$

Next, we calculated the difference between the possible highest and lowest outcomes of the two options as the index of expected regret/relief. This calculation was based on the assumption that the difference between the obtained outcome and the possible outcome if one chose differently would cause the participant’s regret or relief. The bigger the difference, the more intense the regret or relief. R > 0 indicates lower regret/relief from option 1:

(3) $$R=\left(y_{1}-x_{2}\right)-\left(y_{2}-x_{1}\right)$$

The probability of choosing option 1 for each trial of each participant (t, trail number; i, participant number) was calculated as:

(4) $$P\left(O_{1\mathrm{ti}}\right)=1-P\left(O_{2\mathrm{ti}}\right)=F\left({\mathrm{ED}}_{\mathrm{ti}}{\mathrm{,R}}_{\mathrm{ti}}{\mathrm{,EV}}_{\mathrm{ti}}\right)$$

F denotes the inverse logit function to estimate individual expected value, risk variance, and regret. The probability of choosing option 2 was modeled in the same way. We used a linear mixed effect (LME) logistic regression model in R, with EV, ED, and R as continuous fixed-effect factors, the group as a fixed-effect factor, the participant as a random-effect factor, and choice as the binary outcome variable. Another LME logistic regression model was built to test the main effects and interactions among three estimated parameters and groups. Besides the full model, we built multiple models by reducing factors stepwise to check the factor contribution. Likelihood ratio tests were used to confirm statistical significance when comparing models with and without terms of interest. The results were regarded as significant at *p < 0.05*. The criteria to find the best model was the AIC (Akaike Information Criterion) value for each model (39).

To further clarify interactions of suicidal severity with three estimated parameters, we did a sensitivity analysis by building a model with SSI scores at the worst point and EV, ED, and R as continuous fix-effect factors, participant as a random-effect factor, and choice as the outcome variable.

To control for the possible effect of a depressive state, anxiety state and trait, impulsivity, hopelessness, rumination, and childhood maltreatment on the task, we set them as covariates. To check the collinearity of our task parameters, we tested correlations between the slopes of the task parameters and these covariates. Furthermore, to test the effect of change-of-mind, we calculated the frequency of change and repeated the analysis on the rating from the complete feedback by including binary factor (change or not change) in the model as an interaction term.

## Results

### Sample characteristics

There were 202 participants who completed the questionnaires. To match the depressive and anxious levels, rumination, hopelessness, impulsivity, and experience of childhood trauma (CTQ) between the two groups, we excluded 36 healthy controls and 5 suicidal ideators with BDI scores above 14. In total, 80 participants who reported having suicidal ideation at their worst point in life (45 females, age 19.96 ± 1.36) were grouped as individuals with a suicidal disposition (ISD). The mean suicidal ideation score was 14.24 (SD = 7.09). Furthermore, 16 participants had past suicidal attempts (8 females, age 20.56 ± 1.03). Suicidal attempters were different from suicidal ideators in suicidal intention scores (*t* = 5.53, *p* < 0.05). Finally, 79 individuals without any suicidal disposition or psychiatric problems were grouped as controls (HC) (Demographics, Table 1).

**Table 1:** Demographics description of participants.

Group	ISD	HC	*t*	Chi-square	*P* value
Sample size (*N)*	80	79	–	–	–
Age *Mean (SD)*	19.96 (1.36)	20.14 (1.52)	−0.77	–	0.44
Sex (male/female) (*N)*	35/45	39/40	–	0.50	0.53
Scale for Suicide Ideation_worst *Mean (SD)*	14.24 (7.09)	2.37 (4.48)	12.61	–	<0.05*
Scale for Suicide Ideation_current *Mean (SD)*	2.29 (3.84)	0.67 (1.80)	3.29	–	0.05*
Suicide attempts *(yes/no)*	16/64	0/0	–	–	-
BDI *Mean (SD)*	9.93 (7.08)	8.20 (6.32)	1.62	–	0.11
S-AI *Mean (SD)*	40.43 (10.12)	39.91 (9.35)	0.33	–	0.74
T-AI *Mean (SD)*	44.64 (9.29)	43.47 (8.34)	0.84	–	0.41
BIS *Mean (SD)*	59.83 (8.93)	60.00 (7.49)	−0.13	–	0.89
BHS *Mean (SD)*	5.81 (3.04)	5.67 (3.64)	0.23	–	0.79
CTQ *Mean (SD)*	40.63 (11.44)	39.04 (11.11)	0.89	–	0.38
RRS *Mean (SD)*	47.60 (9.73)	45.75 (7.07)	1.38	–	0.17

ISD, individuals with suicidal dispositions; HC, healthy controls; BDI, Beck Depression Inventory; RRS, Rumination Reconsidered scales; S-AI, Spielberger’s State anxiety inventory; T-AI, Spielberger’s State anxiety inventory; BIS, Barratt impulsiveness scale; CTQ, Childhood Trauma Questionnaire.

* *p* < 0.05.

### Disappointment affect ratings

**Figure 2:**
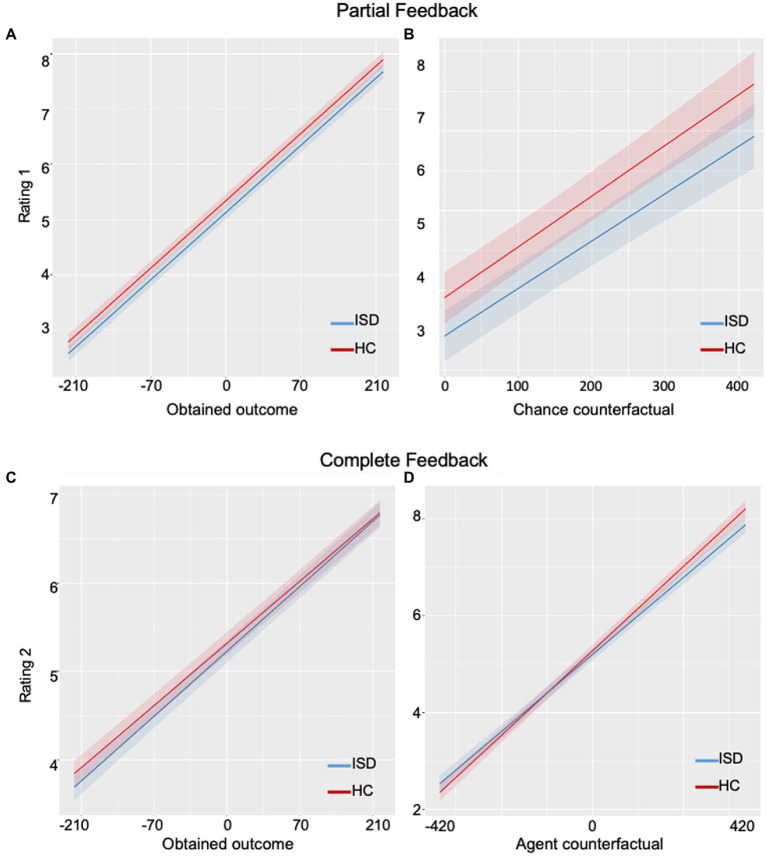
Plots of affective ratings on partial and complete feedback in the suicidal group and the healthy group. **(A)** The rate of disappointment/pleasure upon obtained outcome was not significantly different between groups. **(B)** The rate of disappointment/pleasure upon chance counterfactual outcome was not significantly different between the two groups. **(C)** The rate of regret/relief upon obtained outcome was not significantly different between groups. **(D)** The rate of regret/relief upon agent counterfactual outcome was significantly different between groups: the suicidal group showed blunted responses compared to the healthy group. ISD, individuals with suicidal dispositions; HC, healthy controls.

There was a significant main effect of the group on affective responses to partial feedback. Additionally, a main effect of the obtained outcome on affective responses to partial feedback was also found, with a low obtained outcome related to negative affect and a high obtained outcome related to positive affect in both groups (Figure 2A). We also found a main effect of chance counterfactual on affective responses to partial feedback, with a larger obtained outcome than counterfactual outcome associated with a more positive affect, and a lower obtained outcome than counterfactual outcome associated with a more negative affect (Figure 2B). There was no interaction between the group and obtained outcomes or interaction between the group and chance obtained outcomes to partial feedback (Table 2).

**Table 2:** Affect rating model with obtained and counterfactual outcome parameters.

Parameter	Coefficient	Standard error	95%CI	*t*	*p*
*Affect rating1 model with all subjects*					
Intercept	5.01	6.49 × 10^−2^	4.88 to 5.14	77.24	<0.05*
Obtained outcome	1.22 × 10^−2^	1.83 × 10^−4^	1.18 × 10^−2^ to 1.25 × 10^−2^	66.48	<0.05*
Chance counterfactuals	2.56 × 10^−3^	1.91 × 10^−4^	2.18 × 10^−3^ to 2.93 × 10^−3^	13.37	<0.05*
Group	−1.91 × 10^−1^	9.16 × 10^−2^	−3.71 × 10^−2^ to −1.13 × 10^−2^	−2.09	0.04*
Obtained outcome:group	−2.28 × 10^−5^	2.60 × 10^−4^	−5.33 × 10^−4^ to 4.87 × 10^−2^	−0.09	0.93
Chance counterfactuals:group	−1.70 × 10^−4^	2.71 × 10^−4^	−6.70 × 10^−4^ to −3.61 × 10^−4^	−0.63	0.53
*Affect rating2 model with all subjects*					
Intercept	5.07	6.45 × 10^−2^	5.07 to 5.32	80.57	<0.05*
Obtained outcome	7.03 × 10^−3^	1.70 × 10^−3^	6.70 × 10^−3^ to 7.36 × 10^−3^	41.44	<0.05*
Agent counterfactuals	6.98 × 10^−3^	1.52 × 10^−4^	6.68 × 10^−3^ to 7.36 × 10^−3^	45.86	<0.05*
Group	−7.91 × 10^−2^	9.09 × 10^−2^	−2.57 × 10^−1^ to −9.90 × 10^−2^	−0.87	0.39
Obtained outcome:group	3.09 × 10^−4^	2.39 × 10^−4^	−1.60 × 10^−4^ to 7.78 × 10^−4^	1.29	0.20
Agent counterfactuals:group	−6.08 × 10^−4^	2.15 × 10^−4^	−1.03 × 10^−3^ to −1.88 × 10^−4^	−2.83	<0.05*

* *p* < 0.05.

**Figure 3:**
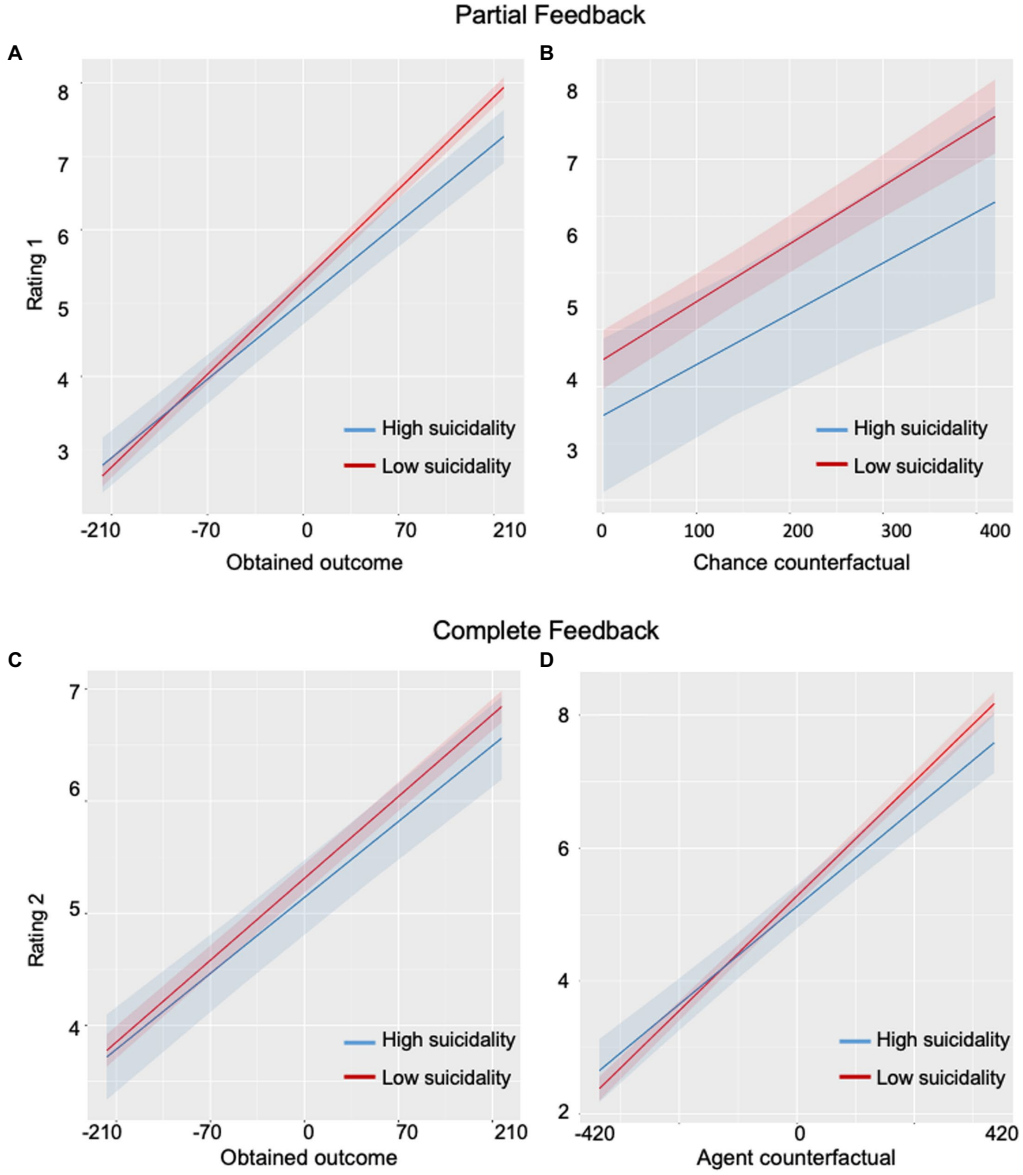
Plots of correlations between levels of suicidal ideation and emotional responses. **(A)** A significant correlation was observed between levels of suicidal ideation and rate of disappointment/pleasure upon obtained outcome: individuals with a high level of suicidal ideation showed less affect than low-suicidality individuals. **(B)** No correlation was found between levels of suicidal ideation and rate of disappointment/pleasure upon chance counterfactual outcome. **(C)** No correlation was found between levels of suicidal ideation and rate of regret/relief upon obtained outcome. **(D)** A significant correlation was found between levels of suicidal ideation and rate of regret/relief upon agent counterfactual outcome.

To examine the effect of suicidal severity on affect ratings to partial feedback, we did a sensitivity analysis by setting scores of Scales for Suicidal Ideation (SSI) as a continuous fix-effect predictor instead of the group with participants with suicidal dispositions. A significant main effect for the obtained outcome (Beta = 1.23 × 10^−2^, SE = 5.95 × 10^−4^, 95%CI = 1.11 × 10^−2^ to 0.0135, *t* = 20.71, *p* < 0.001) as well as a significant interaction between the obtained outcome and the scores for suicidal ideation (Beta = −1.15 × 10^−4^, SE = 3.71 × 10^−5^, 95%CI = -1.88 × 10^−4^ to −4.26 × 10^−5^, *t* = −3.11, *p* = 0.002) were found (Figure 3A). A significant main effect for chance counterfactuals was also found (Beta = 2.049 × 10^−2^, SE = 3.254 × 10^−4^, 95%CI = 0.01 to 0.03, *t* = 6.30, *p* < 0.001). No main effect for suicidal scores was found (Beta = 1.076 × 10^−2^, SE = 8.36 × 10^−3^, 95%CI = -5.62 × 10^−3^ to 2.71 × 10^−2^, *t* = 1.29, *p* = 0.20) nor interaction between chance counterfacutals and suicidal ideations (Beta = −3.84 × 10^−6^, SE = 2.03 × 10^−5^, 95%CI = −4.36 × 10^−5^ to 3.60 × 10^−5^, *t* = −0.19, *p* = 0.85; Figure 3B).

Overall, for partial feedback, the ISD group showed blunted emotions compared to HC participants (Figures 2A,B). Within the ISD group, we found that individuals with higher suicidal ideation scores (high-suicidality) had a more blunted affect to the obtained outcomes than individuals with lower suicidal ideations (low-suicidality; Figure 3A).

### Regret affect ratings

We observed significant main effects of obtained outcome and agent counterfactual on affect rating to complete feedback across all groups (Figure 2C), with a low obtained outcome associated with a stronger negative affect and a high obtained outcome associated with a stronger positive affect. A larger obtained than unobtained outcome was associated with a stronger positive affect and a lesser obtained than unobtained outcome was associated with a stronger negative affect. There was also a significant interaction between the group and agent counterfactuals (Figure 2D). No effect of the group or interaction between the group and obtained outcome were observed (Table 2).

For the sensitivity analysis within the suicidal group, we set the model by adding SSI as a continuous fix-effect factor. Main effects of obtained outcome (Beta = 7.21 × 10^−3^, SE = 3.87 × 10^−4^, 95%CI = 6.45 × 10^−3^ to 7.97 × 10^−3^, *t* = 18.65, *p* < 0.001) and agent counterfactual (Beta = 7.33 × 10^−3^, SE = 3.47 × 10^−4^, *t* = 21.12, *p* < 0.001) were found. A significant interaction between agent counterfactuals and SSI scores was also observed (Beta = −6.68 × 10^−5^, SE = 2.20 × 10^−5^, 95%CI = -1.09 × 10^−4^ to −2.37 × 10^−5^, *t* = −3.04, *p* = 0.002; Figure 3D). No effect of SSI scores (Beta = 6.81 × 10^−3^, SE = 9.24 × 10^−3^, 95%CI = -1.13 × 10^−2^ to 2.49 × 10^−2^, *t* = 0.74, *p* = 0.46) or interaction between SSI and obtained outcome (Beta = 8.66 × 10^−6^, SE = 2.44 × 10^−5^, 95%CI = -3.88 × 10^−3^ to 5.69 × 10^−5^, *t* = 0.36, *p* = 0.72) were found (Figure 3C).

In summary, for complete feedback, the ISD group showed less pleasure than HC when the obtained outcome was larger than the unobtained outcome on the other wheel, and less regret when the obtained outcome was less than the unobtained outcome on the other wheel (Figure 2D). Within the ISD group, high-suicidality individuals showed a more blunted affect to agent counterfactuals than low-suicidality individuals (Figure 3D).

### Decision-making

**Figure 4:**
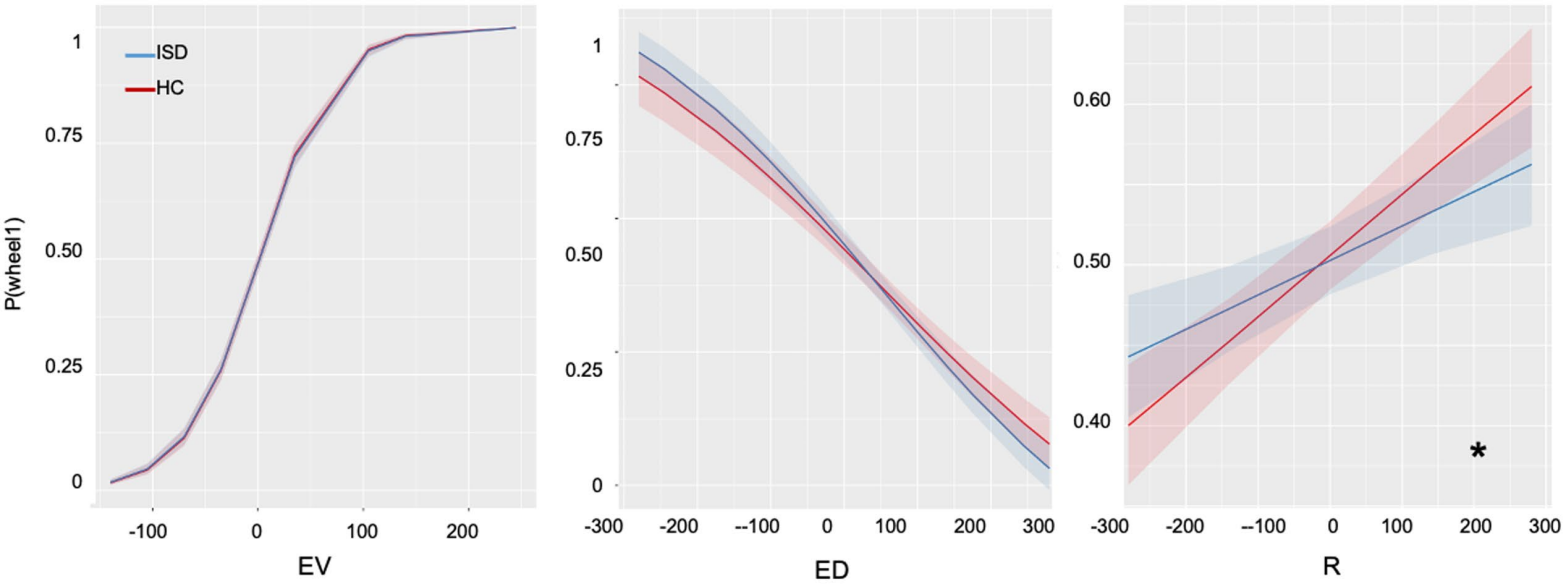
Plots on results of decision-making variables. EV, expected values; ED, expected disappointment; R, avoidance of regret. * indicating *p* < 0.05.

The Effects of the full choice model and the best choice model with computational parameters are summarized in Table 3. We observed a significant main effect of expected value (EV) and a significant main effect of avoidance of disappointment (ED). There was also a significant main effect of regret prediction (R), with participants choosing options to minimize future regret. Importantly, there was a significant interaction between the group and regret prediction. Specifically, the suicidal group showed a blunted sensitivity to future regret compared to healthy controls (Figure 4). There was no interaction between the group and the expected value or avoidance of disappointment parameters.

To examine the effect of suicide severity, we built another linear mixed-effect model with SSI scores as a continuous fixed factor within the ISD group. We found a significant effect for EV (Beta = 0.03, SE = 0.01, z = 9.56, *p* < 0.001), ED (Beta = −4.39 × 10^−3^, SE = 8.38 × 10^−4^, z = −5.23, *p* < 0.001) and R (Beta = 0.01, SE = 5.07 × 10^−4^, z = 4.22, *p* < 0.001) in suicidal individuals. There was also a marginally significant interaction between SSI score and anticipation of future regret (R; Beta = −8.89 × 10^−5^, SE = 3.18 × 10^−5^, z = 2.80, *p* = 0.05), indicating that individuals with a high suicidal disposition are less sensitive to future regret than those with a low suicidal disposition.

In the correlation between task parameters and covariates, we observed a correlation between chance counterfactuals and impulsivity scores, further analysis with BIS as a covariate factor in the linear mixed effect model did not change the result of interaction between chance counterfactual and group (Beta = 6.02 × 10^−5^, SE = 1.14 × 10^−5^, 95%CI = -7.69 × 10^−5^ to −3.35 × 10^−5^, *t* = −2.14, *p* = 0.03). Although significant correlations can be found within covariates or within task variables, no significant correlations were found between task parameters and other covariates (Table 4). Because the three task parameters, ED, EV, and R, were inter-correlated with each other, we checked the variance inflation factors (VIF) in the LME model. The VIFs were all below the commonly suggested cut-off of 10 (VIF values were smaller when the variables were stratified), indicating that collinearity was not a problem in our model (EV:2.85, ED:4.17, R:4.18). The change-of-mind setting did not exacerbate emotional responses to the obtained outcomes (*p* = 0.54) or agent counterfactual (*p* = 0.27). Moreover, although participants had the opportunity to change their minds, very few of them did so, and even then quite infrequently (ISD group: mean = 1.87, SD = 2.73; HC group: mean = 1.94, SD = 2.28). No difference between the groups in the switching wheel rate was found (*t* = 0.16, *p* = 0.87).

**Table 3:** Choice models with computational parameters.

Parameter	Coefficient	Standard Error	95%CI	*t*	*p*
*(A) The best choice model with all subjects*: *choice ~ E + D + R + group:D + group:R + (1 | subject)*					
Intercept	5.16 × 10^−1^	5.80 × 10^−3^	0.50 to 0.53	88.93	<0.05*
EV	3.56 × 10^−3^	8.83 × 10^−5^	3.38 × 10^−3^ to 3.72 × 10^−3^	40.25	<0.05*
ED	−6.06 × 10^−4^	5.99 × 10^−5^	−7.23 × 10^−4^ to −4.88 × 10^−4^	−10.11	<0.05*
R	5.24 × 10^−4^	4.02 × 10^−5^	4.45 × 10^−4^ to 6.03 × 10^−4^	13.02	<0.05*
ED:group	−1.28 × 10^−4^	7.67 × 10^−5^	−2.78 × 10^−4^ to 2.23 × 10^−5^	−1.67	0.10
R:group	−1.19 × 10^−4^	5.12 × 10^−5^	−2.20 × 10^−4^ to −1.90 × 10^−5^	−2.33	0.02*
159 subjects, 12,720 observations					
*(B) Full choice model with all subjects: choice ~ E + D + R + group:E + group:D + group:R + (1 | subject)*					
Intercept	5.16 × 10^−1^	5.80 × 10^−3^	0.50 to 0.53	88.91	<0.05*
EV	3.51 × 10^−3^	1.25 × 10^−4^	3.26 × 10^−3^ to 3.75 × 10^−3^	28.11	<0.05*
ED	−5.95 × 10^−4^	6.33 × 10^−5^	−7.19 × 10^−4^ to −4.71 × 10^−4^	−9.40	<0.05*
R	5.33 × 10^−4^	4.34 × 10^−5^	4.48 × 10^−4^ to 6.18 × 10^−4^	12.27	0.59
ED:group	−1.50 × 10^−4^	8.68 × 10^−5^	−3.20 × 10^−4^ to 2.22 × 10^−5^	−1.74	0.08
R:group	−1.37 × 10^−4^	6.06 × 10^−5^	−2.56 × 10^−4^ to −1.80 × 10^−5^	−2.26	0.02*
159 subjects, 12,720 observations					

EV, expected values; ED, expected disappointment; R, avoidance of regret.

* *p* < 0.05.

## Discussion

In the present study, we examined the association of suicidal ideation with regret anticipation and counterfactual emotional experience in value-based decision-making using model-based mathematical computations. Our results revealed that young adults with suicidal ideation showed a blunted anticipation of potential future regret when making decisions. Whereas suicidal ideators and past suicidal attempters showed less avoidance of future regret, young adults with more suicidal dispositions showed blunted emotional responses to the immediate outcome, regardless of win or loss (Table 4). They were also less sensitive to regret and relief in retrospective comparisons compared to healthy individuals. These results were independent of the state of depression or anxiety, the experience of childhood trauma, ruminations, hopelessness, and impulsivity, which are common risk factors for suicidality and may influence decision-making. Taken together, these findings suggest that subclinical individuals with suicidal dispositions may have specific alterations in the use of forward prospective cognition in action-outcome comparisons to guide goal-directed behaviors and blunted emotional responses to retrospective regret cues.

**Table 4:** Correlations among task parameters and covariates.

	Chance *CF*	Agent *CF*	ED	EV	R	BDI	RRS	SAI	TAI	BIS	CTQ
Chance *CF*	1	0.00*	0.79	0.25	0.33	0.99	0.34	0.69	0.77	0.01*	0.71
Agent *CF*		1	0.61	0.19	0.23	0.23	0.21	0.74	0.86	0.20	0.86
ED			1	0.00*	0.00*	0.27	0.40	0.82	0.51	0.86	0.21
EV				1	0.03*	0.88	0.57	0.33	0.77	0.12	0.47
R					1	0.43	0.42	0.65	0.75	0.42	0.57
BDI						1	0.00*	0.00*	0.00*	0.02*	0.35
RRS							1	0.00*	0.00*	0.11	0.18
SAI								1	0.00*	0.00*	0.02*
TAI									1	0.00*	0.00*
BIS										1	0.05*
CTQ											1

CF, counterfactuals; BDI, Beck Depression Inventory; RRS, Rumination Reconsidered scales; STAI, Spielberger’s State–Trait anxiety inventory; BIS, Barratt impulsiveness scale; CTQ, Childhood Trauma Questionnaire; EV, expected values; ED, expected disappointment; R, avoidance of regret.

* *p* < 0.05.

In healthy individuals, anticipated regret has been suggested to guide decisions that protect one from painful consequences (23). an early clinical study has reported that psychiatric patients with suicidal behaviors have difficulty predicting the consequences or the future value of their behaviors (40) and have then been shown to have deficits in future orientation (41, 42). It has been shown that patients with lesions in the vmPFC have impairments in predicting negative outcomes, learning from negative experiences (43), and avoiding future regret (44). Moreover, dysfunctional value representation in the vmPFC has been observed in suicidal individuals, as has disrupted vmPFC-frontoparietal connectivity in reinforcement learning (45). Taken together, these findings suggest that vmPFC dysfunction might be associated with deficits in regret anticipation in suicidal individuals, including less avoidance of future regret and less consideration of negative consequences, facilitating suicidal behaviors. Our findings in subclinical suicidal ideators confirm that this disrupted anticipation of future-oriented regret might be associated with the severity of suicidality and is independent of co-existing psychiatric disorders.

Individuals with suicidal ideations showed less pleasure in winning and less disappointment in losing, retrospectively, compared to healthy individuals. This may indicate altered value comparisons and amotivated responses to retrospective outcomes. Previous research has found that more than half of suicidal attempters regret their suicidal actions (21). More importantly, the presence of subsequent counterfactual thinking (i.e., wishing that they had died *via* the suicidal acts) is predictive of eventual suicide (46). Although suicidal ideators did not show reduced emotional responses to immediate outcomes, analysis within the ISD group indicated decreased responses to immediate outcomes in individuals with high suicidal severity. It has been shown that suicidal individuals tend to selectively neglect decision-relevant value information in reward learning (29). Impaired value comparison in suicidal individuals has also been found in gambling and reinforcement learning (9, 10, 47, 48). Deficits in consummatory pleasure have been associated with suicide risk (49). Loss of interest and pleasure has been reported to be predictive of suicidal ideation independently of depression in both patients (50) and college students (51, 52). Extending previous findings, our findings of blunted experience of pleasure/relief with positive consequences as well as blunted experience of disappointment/regret with negative consequences in suicidal youths suggest that suicidal disposition might be associated with loss of motivation and flat emotion. These amotivational abnormalities may contribute to an altered value comparison between suicidal behavior and its alterations during a crisis and may potentially increase the likelihood of suicidal behavior.

Suicidal youths did not show a disturbed expected value or altered avoidance of disappointment compared to healthy youths. This might be because we controlled for the level of hopelessness between groups, which is associated with value comparison and despair. However, it has been proposed that negative future expectations, lack of general motivation, and impaired attribution of meanings to personal experiences are key components of hopelessness, which is a strong predictor of suicidal behaviors (53). People with negative expectations about the future and loss of motivation have been reported to have dysfunctions in striatal dopamine pathways, which may affect suicidal ideation (54, 55). Moreover, recent studies have also reported that the absence of positive expectations about the future rather than the global construct of hopelessness, plays a key role in suicidality (56). Therefore, our findings on blunted disappointment in the face of poorer current outcomes, as well as an intact ability to avoid future disappointment may alternatively suggest that this dissociation plays a key role in suicidal disposition.

There are limitations to our study that need to be taken into account in the interpretation of the results. First, given that our participants were recruited from university and that the subclinical suicidal group only shows lifetime suicidal behaviors, the generalization of our results needs to be cautious and our findings need to be replicated in samples of different ages and in psychiatric patients with suicidal dispositions. Second, although we have excluded participants with any diagnosis of mental disorders by an explicit question, a formal diagnosis will be preferred in a future study to control for the undiagnostic risks. Finally, given that both the experience of regret and the prediction of regret might be associated with key areas such as the orbital-frontal cortex and ventromedial prefrontal cortex, future neuroimaging studies are needed to examine neural differences underlying regret processing in suicide.

To conclude, our results suggest that a flat experience and blunted regret prediction are important characteristics in subclinical young adults with lifetime suicidal ideations. These model-based distinctive abnormalities of disappointment experience, regret experience, and regret prediction may shed light on putative trans-diagnostic mechanisms in the early stages of suicidality and may be of help to identify measurable markers of suicidal vulnerability and future intervention targets.

## Data availability statement

The raw data supporting the conclusions of this article will be made available by the authors, without undue reservation.

## Ethics statement

The studies involving human participants were reviewed and approved by Ethics committee of Center for Brain Disorders and Cognitive Sciences, Shenzhen University. The patients/participants provided their written informed consent to participate in this study.

## Author contributions

PX, LD, AA, and YL contributed to the conception and design of the study. HA and LH performed the experiment. HA and LD performed the statistical analyses. HA and PX wrote the draft of the manuscript. All authors contributed to the manuscript revision, and read and approved the submitted version.

## Funding

This study was supported by National Natural Science Foundation of China (Nos. 31700959, 31871137, and 31920103009), Young Elite Scientists Sponsorship Program by China Association for Science and Technology, Zhujiang Talent Project for Postdoctoral researchers, Guangdong International Cooperation and Exchanges (No. 2019A050510048), Guangdong Young Innovative Talent Project (No. 2016KQNCX149), Shenzhen-Hong Kong Institute of Brain Science-Shenzhen Fundamental Research Institutions (No. 2023SHIBS0003), Shenzhen Science and Technology Research Funding Program (Nos. JCYJ20210324093208021, JCYJ20180507183500566, JCYJ20150729104249783, and JCYJ20170412164413575), and Sanming Project of Medicine in Shenzhen (SZSM202003006).

## Conflict of interest

The authors declare that the research was conducted in the absence of any commercial or financial relationships that could be construed as a potential conflict of interest.

## Publisher’s note

All claims expressed in this article are solely those of the authors and do not necessarily represent those of their affiliated organizations, or those of the publisher, the editors and the reviewers. Any product that may be evaluated in this article, or claim that may be made by its manufacturer, is not guaranteed or endorsed by the publisher.

## Footnotes

^1^ http://psychtoolbox.org/
